# BREVIPEDICELLUS Positively Regulates Salt-Stress Tolerance in *Arabidopsis thaliana*

**DOI:** 10.3390/ijms24021054

**Published:** 2023-01-05

**Authors:** Huixian Cai, Yang Xu, Kang Yan, Shizhong Zhang, Guodong Yang, Changai Wu, Chengchao Zheng, Jinguang Huang

**Affiliations:** 1 State Key Laboratory of Crop Biology, College of Life Sciences, Shandong Agricultural University, Tai’an 271018, China; 2 Shandong Peanut Research Institute, Shandong Academy of Agricultural Sciences, Qingdao 266034, China

**Keywords:** arabidopsis thaliana, salt stress, BREVIPEDICELLUS, *XTH7*, xyloglucan

## Abstract

Salt stress is one of the major environmental threats to plant growth and development. However, the mechanisms of plants responding to salt stress are not fully understood. Through genetic screening, we identified and characterized a salt-sensitive mutant, *ses5* (*sensitive to salt 5*), in *Arabidopsis thaliana*. Positional cloning revealed that the decreased salt-tolerance of *ses5* was caused by a mutation in the transcription factor BP (BREVIPEDICELLUS). *BP* regulates various developmental processes in plants. However, the biological function of BP in abiotic stress-signaling and tolerance are still not clear. Compared with wild-type plants, the *bp* mutant exhibited a much shorter primary-root and lower survival rate under salt treatment, while the *BP* overexpressors were more tolerant. Further analysis showed that BP could directly bind to the promoter of *XTH7* (*xyloglucan endotransglucosylase/hydrolase 7*) and activate its expression. Resembling the *bp* mutant, the disruption of *XTH7* gave rise to salt sensitivity. These results uncovered novel roles of BP in positively modulating salt-stress tolerance, and illustrated a putative working mechanism.

## 1. Introduction

Ever-increasing soil salinization is one of the major environmental stresses that severely hampers plant (especially glycophytes) growth, yield and distribution worldwide. Nowadays, more than 20 million hectares of cultivated lands are affected by salt stress [1]. Due to their sessile nature, plants have to absorb excessive ions (mainly Na^+^ and Cl^−^) under high salinity conditions, which imparts a plethora of harms to plants, such as osmotic stress, ion imbalance, oxidative toxicity, and sometimes even cell death [2]. 

From sensing signals to ultimate resistance, plants have evolved highly sophisticated mechanisms to cope with salt stress. Upon detecting salt stress through the sensors in the plasma membrane, e.g., monovalent-cation sensor glycosyl inositol phosphorylceramide (GIPC) sphingolipids [3], a transit buildup of intracellular Ca^2+^ is triggered, which can be decoded via different sets of downstream proteins. Among them, the Salt Overly Sensitive (SOS) pathway is one of the best-characterized modules [4]. Disruption of any main component of the SOS pathway gives rise to hypersensitivity to salt stress in different plant species [5,6]. Other Na^+^ transporters, including Na^+^/H^+^ exchanger 1 (NHX1), high-affinity K^+^ transporter 1 (HKT1) and vacuolar membrane H^+^-pyrophosphatase (VP), are also important players in ionic homeostasis under saline conditions [7,8,9]. In addition, transcriptome remodeling, cellular structural-dynamics, hormonal adjustment, reactive-oxygen-species scavenging, osmolytes accumulation, and post-translational decoration, are all mobilized to gain a systemic resistance to salt stress [10,11,12,13,14,15,16].

Homeobox genes are a highly conserved transcription-factor family ranging from animals to plants [17,18,19]. Maize *KNOTTED1* (*KN1* or *ZmKN1*), which is involved in meristem maintenance, was the first characterized homeobox gene from plants [19,20]. Homologs of *ZmKN1* were cloned from different plant species and constitute the *KNOTTED*-like homeobox (*KNOX*) family [19]. In *A. thaliana*, the *KNOX* genes are divided into two classes. Class I comprises *KNAT1* (*KNOTTED*-like from *A. thaliana*), *KNAT2*, *SHOOT MERISTEMLESS* (*STM*) and *KNAT6* [21,22,23], while class II contains *KNAT3*, *KNAT4* and *KNAT5* [24]. *KNAT*s play vital roles in arabidopsis development [25,26]. Among them, *KNAT1* is also termed *BREVIPEDICELLUS* (*BP*, (AT4G08150)), as a mutation in *BP* gives rise to short pedicels, downward siliques, a compact inflorescence architecture and an obviously reduced overall-height [27,28]. When *BP* is misexpressed in arabidopsis, the overexpressors display lobed leaves with ectopic meristems [29]. Recent discoveries indicate that BP promotes xylem expansion [30,31]. In addition, BP activates the expression of *Peroxidase 17*, to reduce arabidopsis callus-browning by directly binding to its promoter region [32]. However, the biological functions of *KNAT*s in abiotic stress-signaling and tolerance are still not clear.

Xyloglucan is the most important hemicellulose in the primary cell wall of dicotyledonous and non-gramineous monocotyledonous plants [33]. Xyloglucan plays important roles in the rupture and re-generation of the cell wall and the process of plant growth and development [34,35]. The status of xyloglucan was finely modulated by xyloglucan endotransglucosylase/hydrolase (XTH), a family of enzymes subdivided into xyloglucan endotransglucosylase (XET) and xyloglucan endohydrolase (XEH) [36,37,38]. XTHs can effectively modify the structure of the cellulose–xyloglucan complex, and realize cell-wall remodeling by catalyzing the cleavage and reconnection of xyloglucan molecules [39,40]. Recent studies suggest that *XTHs* are closely related to plant response to environmental stresses. Mutations in arabidopsis *XTH17*, *XTH15* or *XTH31* all displayed higher Al^3+^ tolerance compared with wild-type plants [41,42]. *CaXTH3*, a homologue of *Pepper XTH*, responded to various abiotic stresses, and its overexpression in arabidopsis or tomato significantly improved the tolerance of water deficiency and salt stress [43,44]. Transgenic tobaccos overexpressing the populus *PeXTH* enhanced salt tolerance by the development of leaf succulence [45]. The overexpression of persimmon *DkXTH1* apparently enhanced tolerance to diverse abiotic stresses, and delayed fruit softening in transgenic plants [46]. 

In this study, we identified and characterized a salt-sensitive mutant, *ses5* (*sensitive to salt 5*). Positional cloning and sequence analyses validate that the salt-sensitive phenotype of *ses5* was caused by a mutation in *BP*. In contrast, the overexpression of *BP* obviously enhanced the salt tolerance of transgenic arabidopsis. Furthermore, we also authenticated the fact that *XTH7* (AT4G37800) was a downstream target of BP. Resembling *bp,* the *xth7* mutant exhibited sensitivity to salt stress. Our results uncovered novel roles of *BP*, which positively modulates salt tolerance in arabidopsis.

## 2. Results

### 2.1. Mutant ses5 Was Sensitive to Salt Stress

**Figure 1:**
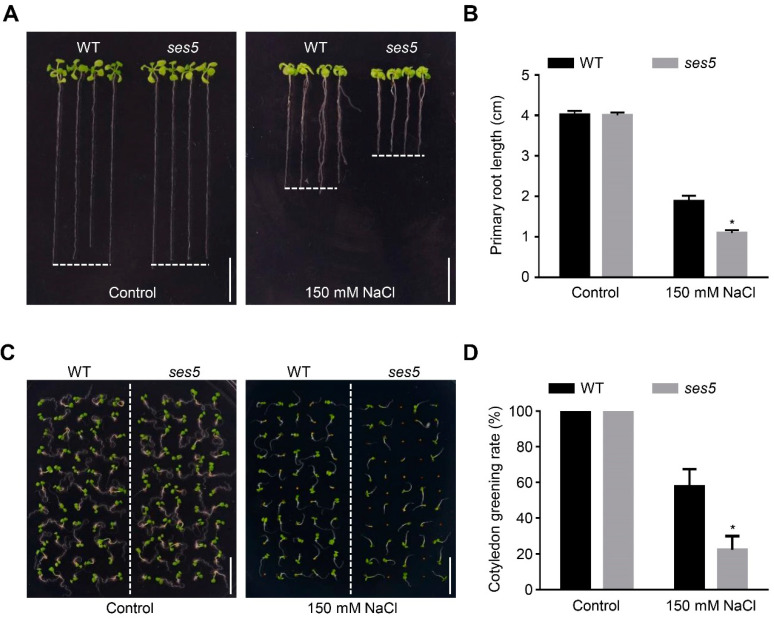
The *ses5* mutant is sensitive to salt stress. (**A**) Photographs of WT and *ses5* seedlings grown vertically on control or salt-containing medium. Three-day-old WT and *ses5* seedlings grown on 1/2 MS medium were transferred to 1/2 MS medium with or without 150 mM NaCl for another 10 d. Scale bar = 1 cm. (**B**) Primary-root length of the seedlings in (**A**). (**C**) Photographs of 7-day-old WT and *ses5* seedlings grown horizontally on 1/2 MS medium with or without 150 mM NaCl. Scale bar = 1 cm. (**D**) Cotyledon-greening rate of the seedlings in (**C**). The bars indicate means ± sd of three independent replicates. * *p* < 0.05 (Student’s *t* test).

To understand the mechanisms adopted by plants to tolerate salt stress, we systematically screened an ethyl methanesulfonate (EMS) mutagenized M_2_ population in the arabidopsis Columbia-0 background via root-elongation assay (Figure S1). One salt-sensitive mutant designated as *ses5* was selected for further study. As shown in Figure 1A, *ses5* seedlings grown on half-strength Murashige and Skoog (MS) plates were indistinguishable from the wild-type (WT) plant. However, when three-day-old seedlings were transferred to medium containing 150 mM NaCl, the *ses5* mutant exhibited a much shorter primary root (Figure 1B). In addition, albeit no divergence in seed-germination rates was observed between *ses5* and WT, regardless of salt stress, the *ses5* mutant showed a significantly lower cotyledon-greening rate under high salinity conditions (Figure 1C,D). These results suggest that the *ses5* mutant was more vulnerable to salt stress.

### 2.2. Positional Cloning of ses5

**Figure 2:**
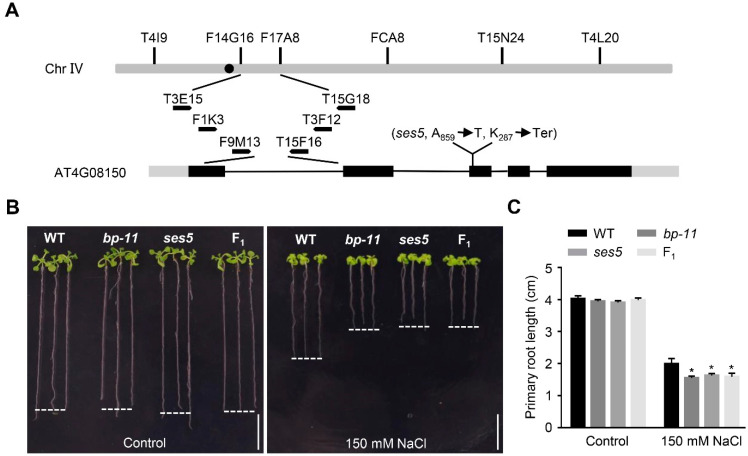
Map-based cloning of *SES5*. (**A**) Genetic mapping and genome structure of *SES5*. Markers used for the genetic mapping are shown on the top. The black box, gray box, and line indicate the exon, the untranslated region, and the intron, respectively. The mutation in *ses5* is shown. (**B**) Photographs of WT, *bp-11*, *ses5*, and F_1_ seedlings grown vertically on control or salt-containing medium. Three-day-old seedlings grown on 1/2 MS medium were transferred to 1/2 MS medium with or without 150 mM NaCl for another 10 d. Scale bar = 1 cm. (**C**) Primary-root length of the seedlings in (**B**). The bars indicate means ± sd of three independent replicates. * *p* < 0.05 (Student’s *t* test).

To identify the gene responsible for the salt-sensitive phenotype of *ses5*, the *ses5* mutant was outcrossed with Landsberg *erecta*, and the F_2_ seedlings displaying sensitivity to NaCl (the sensitive seedlings segregated at a 1:3 ratio, *ses5*:WT = 90:279, χ^2^ = 0.07, *p* > 0.05) were used as the mapping population, following a canonical workflow for mutant-gene identification [47]. The *ses5* mutation was finally mapped to a locus on chromosome IV between bacterial-artificial-chromosome clones F9M13 and T15F16 (Figure 2A). DNA sequencing of candidate genes within this region revealed an A-to-T transversion in the coding region of *BP* (AT4G08150), which generated a premature stop codon (Figure 2A). To validate that the mutated gene was *BP*, we obtained a loss-of-function mutant *bp-11* from the Arabidopsis Biological Resource Center (www.arabidopsis.org, accessed on 16 September 2021). We found that *bp-11* phenocopied *ses5* under salt condition (Figure 2B,C). Similar to *ses5* and *bp-11*, their F_1_ seedlings were also sensitive to salt stress (Figure 2B,C), indicating that *ses5* and *bp-11* are allelic to each other. In addition, the *ses5* displayed downward-pointing siliques (Figure S2A), which was a typical feature observed in *bp* mutants. Therefore, *ses5* was renamed *bp-12*. Moreover, *BP* was apparently up-regulated by salt treatment (Figure S2B,C). Taken together, the salt-sensitive phenotype of *ses5* was caused by the mutation of *BP*, and salt-responsive *BP* is a positive regulator in arabidopsis salt-stress tolerance.

### 2.3. Overexpression of BP Enhances Salt-Stress Tolerance

**Figure 3:**
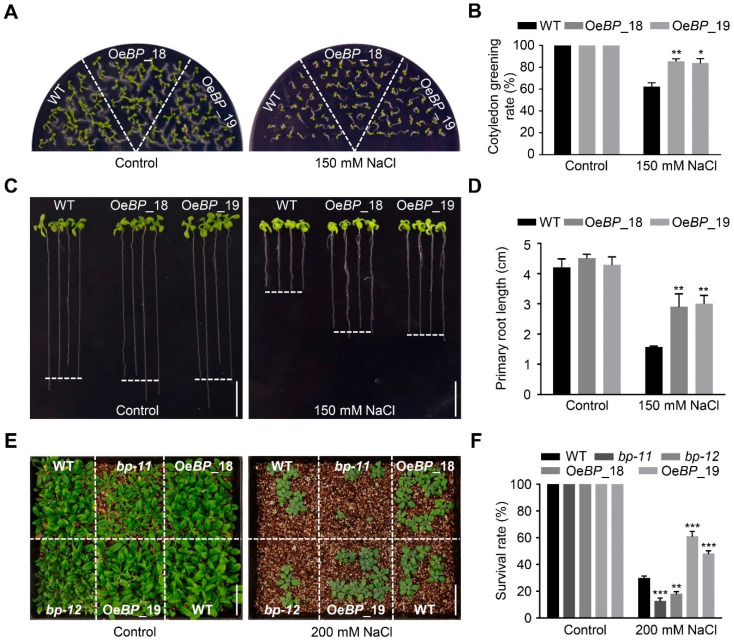
Overexpression of *BP* enhances salt-stress tolerance. (**A**) Seven-day-old WT and *BP*-overexpression seedlings grown on 1/2 MS medium with or without 150 mM NaCl. (**B**) Cotyledon-greening rate of the seedlings in (**A**). (**C**) WT and *BP*-overexpression lines on 1/2 MS medium with or without 150 mM NaCl. Three-day-old seedlings of WT and *BP*-overexpression lines grown on 1/2 MS medium were transferred to 1/2 MS medium with or without 150 mM NaCl for another 10 d. Scale bar = 1 cm. (**D**) Primary-root length of the seedlings in (**C**). (**E**) Four-week-old WT, mutants and overexpression lines grown in vermiculite with or without 200 mM NaCl treatment. Scale bar = 5 cm. (**F**) Survival rate of the seedlings in (**E**). The bars indicate means ± sd of three independent replicates. * *p* < 0.05, ** *p* < 0.01, *** *p* < 0.001 (Student’s *t* test).

As *bp* mutants showed dampened tolerance to salt stress, we reasoned that its overexpression may enhance salt tolerance. To test our hypothesis, transgenic arabidopsis plants overexpressing *BP* driven by the 35S promoter were generated. Among them, two homozygous transgenic lines (Oe*BP*_18 and Oe*BP*_19) with relatively higher expression of *BP* were selected for further analysis (Figure S3A). On half-strength MS medium, there was no noticeable difference between WT and the two transgenic lines. However, in the presence of salt, the transgenic lines exhibited significantly higher cotyledon-greening rates and a longer primary root, compared with WT (Figure 3A–D). In addition, we also investigated the salt responses of WT, *bp* mutants and *BP*-overexpression lines grown in vermiculite. After treatment with 200 mM NaCl solution for two weeks, approximately 30% only of the WT plants survived, whilst more than 50% of *BP*-overexpression plants survived (Figure 3E,F). Salt stress usually causes excessive accumulation of ROS (reactive oxygen species), which will lead to oxidative damage in plant cells. To evaluate the ROS levels under salt treatment, we used 3,3′-diaminobenzidine (DAB) and nitro blue tetrazole (NBT) for histochemical staining. The results indicated that the WT, mutants and overexpression lines accumulated similar low contents of H_2_O_2_ and O_2_^−^ under normal conditions. However, after salt treatment, the mutants accumulated higher levels of H_2_O_2_ and O_2_^−^, while the overexpression lines accumulated less H_2_O_2_ and O_2_^−^, compared with WT (Figure S3B,C). These data support the fact that overexpression of *BP* could remarkably improve the performance of transgenic arabidopsis under salt stress.

### 2.4. BP Transactivates the Expression of XTHs

**Figure 4:**
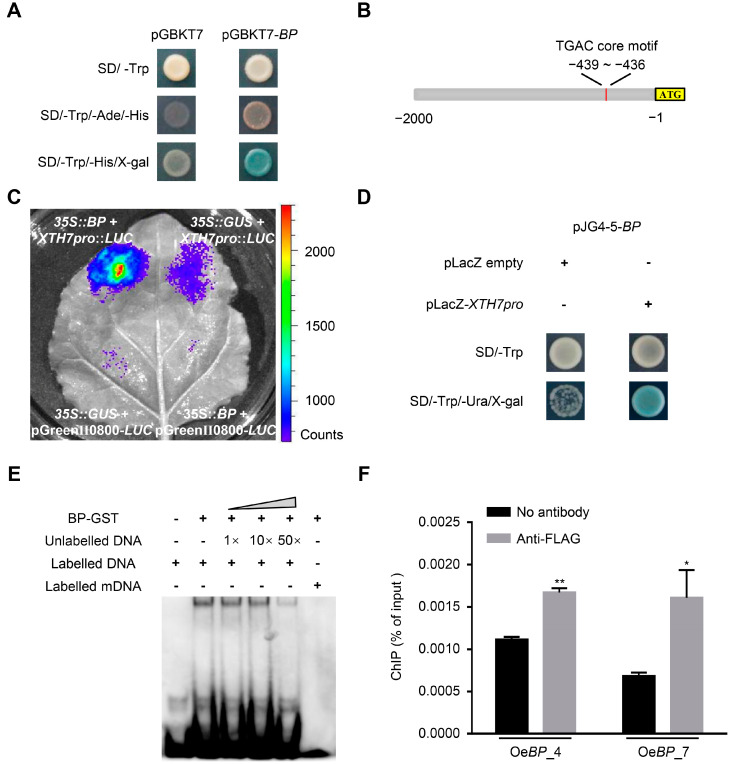
BP directly binds to the promoter of *XTH7*. (**A**) Transcriptional-activity assay of BP. The full length CDS of *BP* was fused in frame to the GAL4 DNA-binding domain and transformed into yeast-strain Gold Yeast. The pGBKT7 vector was used as the negative control. (**B**) Schematic diagram of the promoter of *XTH7*. The TGAC core motif is shown in red. (**C**) BP activates the expression of *XTH7pro:LUC.* The effector (*35S:BP*) and reporter (*XTH7pro:LUC*) vectors were co-transformed into *N. benthamian* leaves. Tobacco leaves injected with pGreenII0800-*LUC* + *35S:GUS*, *XTH7pro:LUC* + *35S:GUS*, pGreenII0800-*LUC* + *35S:BP* were used as controls. (**D**) Yeast one-hybrid assay showing the binding of BP to the promoter of *XTH7*. (**E**) BP binds to the promoter of *XTH7* in EMSA. The probes were labelled with biotin. (**F**) BP combines with the promoter of *XTH7* in the ChIP assay. The bars indicate means ± sd of three independent replicates. * *p* < 0.05, ** *p* < 0.01 (Student’s *t* test).

The biological significance of *BP* in resisting salt stress has not been reported before. To reveal the underlying mechanism, we firstly analyzed the transcriptional activity of BP. As shown in Figure 4A, when GAL4BD-BP was expressed, the yeast strain Gold Yeast could grow and proliferate on SD medium lacking adenine and histidine, indicating that BP possesses transcriptional-activation activity. Based on this observation, the putative target genes of BP were screened using RNA sequencing (RNA-seq) of seven-day-old WT and *BP*-overexpression line Oe*BP*_18 (Figure S4A). Given that BP functions as a transcriptional activator, we mainly focused on the up-regulated genes in *BP*-overexpression plants within the RNA-seq data (Table S1). Gene Ontology analysis of these 268 up-regulated genes (log_2_Fold_Change > 0.5, Padj < 0.05) showed a statistically significant enrichment of categories involved in secondary-metabolite biosynthetic process, the phenylpropanoid biosynthetic and metabolic process, and plant-type cell-wall organization or biogenesis (Figure S4B,C).

Although no marker genes participating in salt stress were detected in our transcriptome data, three members belonging to the xyloglucan endotransglucosylase/hydrolase (XTH) family (*XTH7*, *XTH8* and *XTH15*) showed higher expression in the *BP*-overexpression line (Table S1). Previous studies revealed that BP usually recognizes the *cis*-element harboring the TGAC core motif [48]. Through searching the 2000 bp region upstream of the start codon, only one TGAC core motif was found in the promoter region of *XTH7*, *XTH8* and *XTH15* (Figure 4B and Figure S5A). As *XTH*s were reported to modulate salt stress [43,49], we focused on these three up-regulated *XTH*s in the *BP*-overexpression lines. To substantiate the expression tendency detected by RNA-seq, we re-examined the transcription level of *XTH7*, *XTH8* and *XTH15* by RT-qPCR (Figure S5B–D). We found that only *XTH7* was obviously enhanced in the *BP*-overexpression lines (Figure S5B). In addition, when *35S:BP* and *XTH7pro: Luciferase* (*LUC*) constructs were co-infiltrated into the tobacco leaves, a much stronger fluorescence was observed (Figure 4C). These results suggested that *XTH7* could be trans-activated by BP.

To further determine the recognition of BP on *XTH7*, we examined the binding ability of BP to the TGAC core motif within the promoter region of *XTH7*. The yeast one-hybrid assay showed that BP could bind to the promoter region (26 bp spanning the TGAC core motif) of *XTH7* (Figure 4D). Moreover, in the electrophoretic mobility shift assay (EMSA), a clear band shift was detected, reflecting the formation of the complexes composed of BP and biotin-labelled DNA probes (Figure 4E). In addition, the binding activity to biotin-labelled DNA probes gradually decreased with the addition of unlabeled competitors (Figure 4E). When the core motif TGAC was replaced by AAAA, BP could not bind to the labelled probe anymore (Figure 4E). Furthermore, we performed chromatin immunoprecipitation (ChIP) using a FLAG antibody to assess the DNA-binding activity of BP to the *XTH7* promoter in vivo. Two transgenic lines, Oe*BP*_4 and Oe*BP*_7, were selected for further analysis (Figure S5E). The ChIP-qPCR results indicated that BP could bind to the promoter fragment of *XTH7* containing the TGAC core motif (Figure 4F). These results support the fact that BP directly activates the transcription of *XTH7*, via binding to the TGAC core motif.

### 2.5. Mutant xth7 Is Sensitive to Salt Stress

**Figure 5:**
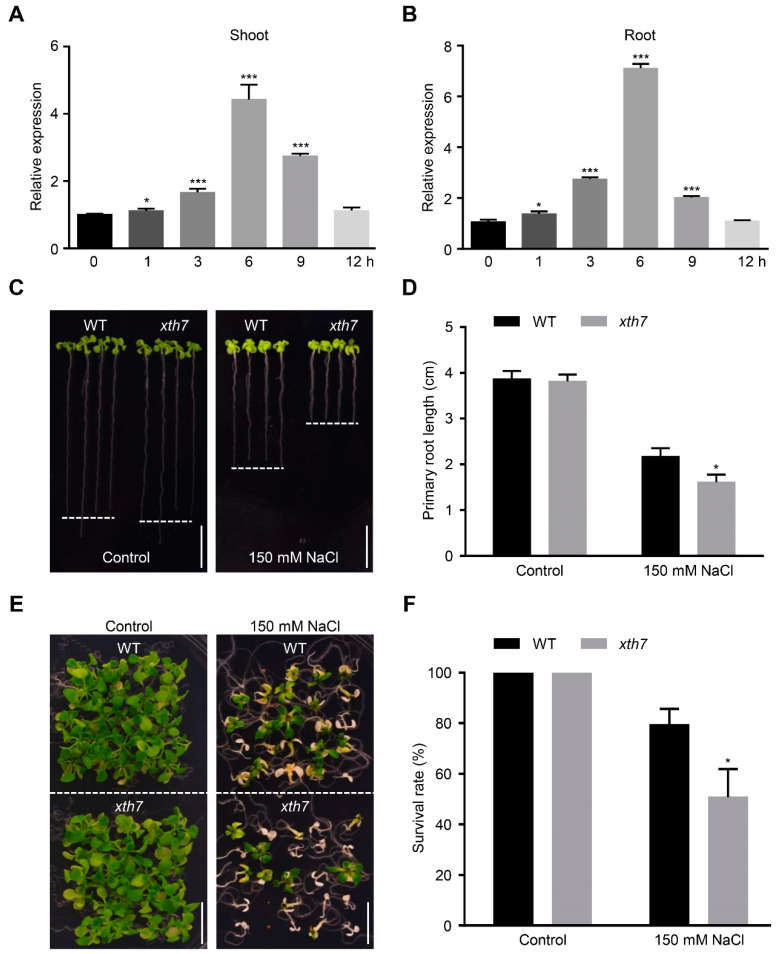
The *xth7* mutant is sensitive to salt stress. (**A**) Responsiveness of shoot *XTH7* to salt stress. (**B**) Responsiveness of root *XTH7* to salt stress. In (**A**,**B**), 7-day-old seedlings were treated with 200 mM NaCl for 0, 1, 3, 6, 9 and 12 h. The data were normalized against the expression of *GAPDH* and *UBQ10*. The bars indicate means ± sd of three independent replicates and are compared with the no-treatment condition (0 h). * *p* < 0.05, *** *p* < 0.001 (Student’s *t* test). (**C**) Photographs of WT and *xth7* seedlings grown vertically on control or salt-containing medium. Three-day-old WT and *xth7* seedlings grown on 1/2 MS medium were transferred to 1/2 MS medium with or without 150 mM NaCl for another 10 d. Scale bar = 1 cm. (**D**) Primary-root length of the seedlings in (**C**). (**E**) Photographs of three-week-old WT and *xth7* seedlings grown on 1/2 MS medium with or without 150 mM NaCl. Scale bar = 1 cm. (**F**) Survival rate of the seedlings in (**E**). The bars indicate means ± sd of three independent replicates. * *p* < 0.05 (Student’s *t* test).

Similarly to *BP*, *XTH7* was also induced by NaCl treatment (Figure 5A,B). Given that *bp* mutants displayed salt sensitivity and that BP could directly activate the transcription of *XTH7* (Figure 1 and Figure 4), we asked whether *XTH7* was involved in salt-stress tolerance. We firstly examined the responses of a knockout mutant of *XTH7* (*xth7*, SALK_201184C) to salt treatment (Figure S6A). As shown in Figure 5C, no visible difference was observed between WT and *xth7* under control conditions. Nevertheless, the root length of *xth7* was much shorter and the survival rate of *xth7* was significantly lower under the NaCl-containing medium (Figure 5C–F). The salt-sensitive phenotype of *xth7* was totally restored by a WT genomic fragment containing *XTH7* and its promoter (Figure S6B,C). However, no visible enhancement in salt tolerance was observed when *XTH7* was overexpressed in arabidopsis (Figure S6D–F). These results show that *XTH7* is required for arabidopsis salt-tolerance.

### 2.6. Mutant of xth7 Contains Lower Xyloglucan

**Figure 6:**
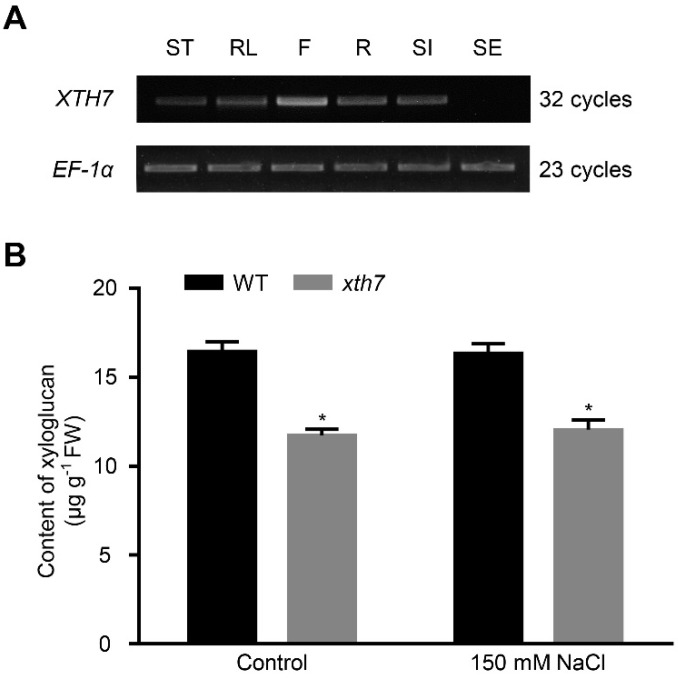
The *xth7* mutant contains lower xyloglucan. (**A**) Expression levels of *XTH7* in different tissues detected by RT-PCR. ST: stem; RL: rosette leaf; F: flower; R: root; SI: silique; SE: seed. (**B**) Extractable xyloglucan content of WT and *xth7* plants with or without 150 mM NaCl treatment. The bars indicate means ± sd of three independent replicates. * *p* < 0.05 (Student’s *t* test).

XTH7 was catalogued in the xyloglucon transglycosylase (XET; EC 2.4.1.207) family [33]. XETs are involved in the depolymerisation of plant structural polysaccharides such as xyloglucan, and in the incorporation of newly synthesized xyloglucan components [50]. As *XTH7* was expressed in all tissues except seeds (Figure 6A), we examined the total xyloglucan content of WT and *xth7* mutant from cell-wall extracts, via iodine staining. The results showed that the extractable xyloglucan-level was reduced by 25% in *xth7* compared to WT under normal or salt-stress conditions (Figure 6B). Consistent with the phenotype, no obvious difference in extractable xyloglucan-content was observed between WT and *XTH7*-overexpression lines (Figure S6G). These results indicate that excess XTH7 is insufficient for improving the salt tolerance of arabidopsis.

## 3. Discussion

BP is well-known for maintaining the function of meristems [22]. Nevertheless, the involvement of BP in salt-stress tolerance has not been illustrated before. In this study, we provided convincing clues that BP was indispensable for salt-stress tolerance in arabidopsis. Contrary to the salt-sensitive phenotype of *bp* mutants, the overexpression of *BP* conferred a significantly higher survival-rate and a longer primary root on transgenic arabidopsis, which may benefit the plants with stronger water-uptake and transportation, and then reduce intracellular Na^+^ concentration under salt-stress condition. *BP* belongs to the *KNOX* gene family, and there are six paralogues of *BP* in *A. thaliana* genome [51,52]. However, their roles in modulating salt stress and/or other stresses are still elusive at present. As *KNOX* genes are highly conserved in plants [24], it is plausible that the counterparts of *BP* in other species may also participate in salt-stress tolerance.

Consistent with previous reports [32], our data showed that BP could act as a transcriptional activator. To uncover the mechanism of BP in adjusting arabidopsis salt-stress tolerance, the putative down-stream targets of BP were screened via RNA-seq analysis. Transcriptomic analyses showed that, compared with WT, there was a statistically significant enrichment of genes involved in the secondary-metabolite biosynthetic process, the phenylpropanoid biosynthetic and metabolic process, and in plant-type cell-wall organization or biogenesis, which may be one of the reasons for enhanced resistance to salt stress. Fortifying cell walls is one of the common defense-mechanisms in plants. It is worth noting that BP also regulates the expression of certain genes involved in boundary development and lignin biosynthesis [53,54], illustrating that the transcription activity of BP is delicately supervised in different cell-types and/or fluctuating environmental conditions.

Previous studies reported that the ectopic expression of *BP* in arabidopsis transforms simple leaves into lobed leaves [29]. However, only increased serration was observed in the leaves of our *BP*-overexpression plants (Figure S7). This discrepancy might arise from different ecotypes and/or different expression-levels of the *BP* transgene. The expression of *BP* was also modulated by auxin [55]. In recent years, many studies have provided evidence to show that auxin is also closely related to abiotic stress resistance. Whether BP modulates salt stress through auxin signaling, remains unknown.

Xyloglucan is the most important hemicellulose in the primary cell-wall of dicotyledonous and non-gramineous monocotyledonous plants [33]. Mutation in arabidopsis *XTH17*, *XTH15* or *XTH31* all displayed higher Al^3+^ tolerance compared with WT plants [41,42]. The *CaXTH3*-over-expressing transgenic arabidopsis or tomato significantly improved the tolerance of water deficiency and salt stress [43,44]. XTH7, screened via RNA-seq analyses, belongs to the subgroup II xyloglucon transglycosylase in *A. thaliana* [33]. One working mechanism for XET is cross-linking cellulose and xyloglucans through hetero-transglycosylaton reactions that enhance tighter packing of the cell wall [56]. Therefore, it is possible that lower expression of *XTH7* in *bp* mutants and the totally lack of *XTH7* in *xth7* all lead to the loosening of cell walls, which facilitates Na^+^ diffusion and uptake, giving rise to much more severe Na^+^-triggered cellular damage.

In addition, environmental stresses trigger ROS accumulation, and lead to oxidative damage [57]. However, plant cells are well equipped with antioxidants and scavenging enzymes to maintain the ROS levels. Through histochemical staining, much lower ROS was detected in *BP*-overexpression lines, indicating that BP could also strengthen the ROS-scavenging system under salt-stress conditions.

## 4. Materials and Methods

### 4.1. Plant Materials and Growth Conditions

*A. thaliana* ecotype Col-0 and L*er* were used. The mutants *bp-11* (CS3161) and *xth7* (SALK_201184C) were obtained from the Arabidopsis Biological Resource Center (http://www.arabidopsis.org, accessed on 16 September 2021). Homozygous T-DNA insertion mutants were identified using PCR-based genotyping with a T-DNA-specific primer and gene-specific primers (Table S2). After surface sterilization with ethanol (70%, 5 min) and NaClO (26% NaClO, 10 min), the seeds were placed in 1/2 MS plates (Sigma-Aldrich, MO, USA; pH 5.8) containing 1.5% (*w*/*v*) sucrose. After stratification at 4 °C for 2 d, the plates were placed in an incubator (22 °C, 16 h light/8 h dark, 120 µmol m^−2^ s^−1^) for germination. Seedlings of approximately seven-days old were transplanted into the soil and grown to maturity in a growth chamber (22 °C, 16 h light/8 h dark, 120 µmol m^−2^ s^−1^).

### 4.2. Mutant Isolation and Genetic Mapping

Mutants were screened on plates containing 150 mM NaCl, according to a previous report [15]. To map the *ses5* mutation, the *ses5* mutant was crossed with *Landsberg erecta*. A total of F_2_ plants exhibiting the salt-sensitive phenotype were selected as a mapping population. Genomic DNA from these F_2_ plants was extracted and used for PCR-based mapping (http://amp.genomics.org.cn/, accessed on 10 June 2021). Genomic-DNA fragments corresponding to candidate genes were PCR amplified from *ses5*, and used in the DNA-sequencing analysis to identify the mutation. Primers are listed in Table S2.

### 4.3. Vector Construction and A. thaliana Transformation

The full-length cDNA of *BP*, full-length cDNA of *XTH7* and genomic DNA fragment of *XTH7* were inserted into the binary vector pBI121 (*35S: BP*), pPZP211 (*35S: BP-3* × *FLAG*), pSuper1300 (*35S: XTH7*) and pMDC107 (*XTH7pro: XTH7*) via digestion and ligation, respectively. These structures were confirmed by sequencing, and then introduced into *Agrobacterium tumefaciens* GV3101. *A. thaliana* was transformed using the floral-dip method [58]. The T_2_ line that produced 100% kanamycin or hygromycin-resistant plants in the T_3_ generation was considered a homozygous transformant. The vector construct images are shown in Figure S8.

### 4.4. Salt Treatment

In the germination experiment, seeds of each genotype were sterilized and sown on 1/2 MS medium with or without salt. Germination was defined as the protrusion of a radicle, and calculated from at least three independent experiments. For root-length analysis, 3-day-old seedlings for each genotype were transferred to 1/2 MS medium with or without 150 mM NaCl for 10 d. In the survival-rate assay, 7-day-old seedlings of each genotype were transplanted into vermiculite. Two weeks later, these plants were watered with nutrient solution or nutrient solution containing 200 mM NaCl (once every 3 d). After treatment for a week, the survival rate was calculated.

### 4.5. RT-PCR and RT-qPCR Analyses

Total RNAs were extracted from 7-day-old seedlings using RNAiso Plus (TaKaRa, Ohtsu, Japan) and 1 μg total RNA was used for reverse transcription using PrimeScript reverse transcriptase with oligo(dT) primer using the Prime Script RT Enzyme MIX I (TaKaRa, Ohtsu, Japan).

To detect relative expression levels, the RNA samples were analyzed using quantitative real-time PCR (RT-qPCR). *EF-1α* was used as the internal control for RT-PCR. Primers are listed in Table S2. The RT-qPCR was performed using the SYBR Green real-time PCR master mix (TaKaRa, Ohtsu, Japan) and the CFX96™ Real-time System (Bio-Rad, Hercules, CA, USA) with the following standard cycling conditions: 95 °C for 10 s, followed by 40 cycles of 95 °C for 5 s and 60 °C for 30 s. The data are presented after normalizing to the reference genes *UBQ10* and *GAPDH*. Each treatment had three biological replicates and three parallel sample-replicates.

### 4.6. RNA-Seq Analysis

Seven-day-old WT and *BP*-overexpression line were treated with 200 mM NaCl for 12 h and then harvested for RNA extraction. The transcriptome analysis was performed by Novogene with three biological repeats. Library construction was performed according to Illumina standard instructions. Reads were aligned to the *A. thaliana* genome, using TopHat2 [59]. Genes with adjusted *p* < 0.05 were considered to be differentially expressed.

### 4.7. Yeast One-Hybrid Assay

The full-length CDS of *BP* without the termination codon was cloned by Phantamax Super-Fidelity DNA Polymerase (Vazyme, Nanjing, China) and inserted into the pJG4-5 vector (Clontech, Mountain View, CA, USA). To prepare a construct for the yeast one-hybrid assay, the promoter region of *XTH7* (−447 to −424) was amplified and cloned into the *Kpn*I and *Sal*I sites in the pLacZi2μ vector, resulting in the *XTH7pro:LacZ* reporter constructs. Recombinant constructs were co-transferred into the yeast strain EGY48 and cultured on SD/−Trp, SD/−Trp/–Ura medium containing X-gal. A yeast one-hybrid analysis was performed according to a previous description [60]. The experiments were performed three times. All primers used here are listed in Table S2.

### 4.8. Transient-Expression Assay

The construct carrying the full-length CDS of *BP* was used as the effector. The *XTH7*-promoter fragment (26 bp) was cloned into the reporter vector pGreenII0800 containing the firefly-*LUC* reporter gene to generate *XTH7pro: LUC*. The recombinant plasmid and control plasmid were transformed into *Agrobacterium tumefaciens* GV3101, respectively. Then, different sets of *Agrobacteria* were co-injected into fully developed leaves of four-week-old *N. benthamiana*. Three days later, D-Luciferin (1 mM) was sprayed onto the tobacco leaves and they were kept in the dark for 5 min. Then photographs were taken using a CCD camera (1300B, Roper, Sarasota, FL, USA) set at −110 °C.

### 4.9. EMSA

The full-length CDS of *BP* was cloned and ligated into the expression vector pGEX4T-3. The recombinant construct was transformed into *Escherichia coli* (Rosetta2). After induction with 0.5 µM isopropyl β-d-1-thiogalactopyranoside, the recombinant BP-GST protein was purified using a GST-tagged purification-resin kit (Beyotime Biotechnology, Shanghai, China). The promoter region of *XTH7* (−447 to −424) was amplified using biotin-labeled primers (Table S2) synthesized by Sangon biotech. The EMSA was conducted using a LightShift Chemiluminescent EMSA kit (Thermo Fisher Scientific, Pierce, CO, USA) following the manufacturer’s protocol.

### 4.10. Western Blot Analysis

Total proteins from *35S: BP-3* × *FLAG* transgenic arabidopsis plants were separated by SDS-PAGE. Proteins were then transferred to the Immobilon-PVDF membrane (Millipore, IPVH00010, Sigma-Aldrich, MO, USA). The membranes were blocked for 1 h in 5% fat-free milk powder dissolved in TBS-Tween, and then incubated with the primary antibodies for 1 h, followed by incubation with the corresponding secondary antibodies for 1 h. The bands were visualized using ECL substrate (NCM Biotech, Suzhou, China). The signals were detected using the Chemiluminescence Imaging System (K4000, KCRX Biotechnology, Beijing, China).

### 4.11. ChIP-qPCR Assay

The ChIP-qPCR assay was performed as described previously [61]. Seven-day-old transgenic seedlings (*35S: BP-3 × FLAG*) were harvested and used for the ChIP assays. The mouse monoclonal FLAG-antibody (TransGen Biotech, Beijing, China) was used. The ChIP DNA products were analyzed using qPCR with primers designed to amplify the DNA fragment in the promoter of *XTH7* (Table S2).

### 4.12. Xyloglucan Content Quantified by Iodine Staining

The fractionation of cell walls was conducted as previously described [62]. Separated tissues were fixed for 10 min in 15 mL of boiling methanol. The methanol-fixed tissues were rehydrated with water, then homogenized in water with a mortar and pestle. The residue obtained by centrifugation was washed with water, acetone, and a methanol:chloroform mixture (1:1, *v*/*v*) and air-dried. The washed residue was dried overnight at 40 °C and then treated with 2 units/mL α-amylase (Macklin, Shanghai, China) in 100 mM MOPS (Coolaber, Beijing, China) buffer (pH 7.3) for 0.5 h at 80 °C, then with 1 unit/mL pullulanase (Coolaber, Beijing, China) and 3 units/mL amyloglucosidase (Coolaber, Beijing, China) in sodium acetate buffer for 3 h at 50 °C, to remove starch. Hemicellulose was extracted for 18 h with 17.5% NaOH containing 0.02% NaBH4. The hemicellulosic fraction was neutralized with glacial acetic acid in an ice-cold water bath, then dialyzed against water. The dialyzed hemiceiiuiosic fraction was centrifuged for 20 min at 10,000× *g*, and dried. The xyloglucan content was determined using the iodine-staining method [62].

### 4.13. ROS Staining

The O_2_^−^ and H_2_O_2_ levels were determined with NBT and DAB staining [63], respectively. Seven-day-old seedlings were treated with or without 150 mM NaCl for 24 h. For NBT staining, the seedlings were vacuum infiltrated for 10 min and then stained for 12 h with 0.05% NBT (*w*/*v*) dissolved in distilled water, at room temperature and in total darkness. For DAB staining, the seedlings were vacuum infiltrated for 10 min and then stained with 0.1% DAB-tetrahydrochloride (*w*/*v*) dissolved in distilled water at room temperature and in total darkness. Subsequently, the seedlings were incubated in de-staining buffer (ethanol:lactic acid:glycerol, 3:1:1) at 80 to 90 °C until colorless, and then mounted in ethanol. Three biological repeats were performed (20 plants per biological repeat), and one of the representative pictures was shown.

## 5. Conclusions

In this study, we identified and characterized a novel role of BP in modulating salt-stress tolerance. The *bp* mutants showed sensitivity to salt stress, while overexpression of *BP* apparently enhanced salt-tolerance. BP directly binds to the TGAC core motif and activates the expression of *XTH7*, which plays an important role in stabilizing the xyloglucan content of the cell wall. These data imply that the dynamic ultrastructure of the cell wall plays an indispensable part in resisting salt stress, and is worthy of further investigation in the future.

## Supplementary Materials

The following supporting information can be downloaded at: https://www.mdpi.com/article/10.3390/ijms24021054/s1.

## References

[1] Wang Y., Cao Y., Liang X., Zhuang J., Wang X., Qin F., Jiang C. (2022). A dirigent family protein confers variation of Casparian strip thickness and salt tolerance in maize. Nat. Commun..

[2] van Zelm E., Zhang Y., Testerink C. (2020). Salt Tolerance Mechanisms of Plants. Annu. Rev. Plant. Biol..

[3] Jiang Z., Zhou X., Tao M., Yuan F., Liu L., Wu F., Wu X., Xiang Y., Niu Y., Liu F. (2019). Plant cell-surface GIPC sphingolipids sense salt to trigger Ca^2+^ influx. Nature.

[4] Zhang H., Zhu J., Gong Z., Zhu J.K. (2022). Abiotic stress responses in plants. Nat. Rev. Genet..

[5] Ji H., Pardo J.M., Batelli G., Van Oosten M.J., Bressan R.A., Li X. (2013). The Salt Overly Sensitive (SOS) pathway: Established and emerging roles. Mol. Plant.

[6] Manishankar P., Wang N., Koster P., Alatar A.A., Kudla J. (2018). Calcium Signaling during Salt Stress and in the Regulation of Ion Homeostasis. J. Exp. Bot..

[7] Apse M.P., Aharon G.S., Snedden W.A., Blumwald E. (1999). Salt tolerance conferred by overexpression of a vacuolar Na^+^/H^+^ antiport in *Arabidopsis*. Science.

[8] Rubio F., Gassmann W., Schroeder J.I. (1995). Sodium-driven potassium uptake by the plant potassium transporter HKT1 and mutations conferring salt tolerance. Science.

[9] Gaxiola R.A., Li J., Undurraga S., Dang L.M., Allen G.J., Alper S.L., Fink G.R. (2001). Drought- and salt-tolerant plants result from overexpression of the AVP1 H^+^-pump. Proc. Natl. Acad. Sci. USA.

[10] Feng W., Kita D., Peaucelle A., Cartwright H.N., Doan V., Duan Q., Liu M.C., Maman J., Steinhorst L., Schmitz-Thom I. (2018). The FERONIA Receptor Kinase Maintains Cell-Wall Integrity during Salt Stress through Ca^2+^ Signaling. Curr. Biol..

[11] Yu Z.P., Duan X.B., Luo L., Dai S.J., Ding Z.J., Xia G.M. (2020). How Plant Hormones Mediate Salt Stress Responses. Trends Plant Sci..

[12] Yang Y., Guo Y. (2018). Elucidating the molecular mechanisms mediating plant salt-stress responses. New Phytol..

[13] Wang C., Zhang L.J., Huang R.D. (2011). Cytoskeleton and plant salt stress tolerance. Plant Signal. Behav..

[14] Prerostova S., Dobrev P.I., Gaudinova A., Hosek P., Soudek P., Knirsch V., Vankova R. (2017). Hormonal dynamics during salt stress responses of salt-sensitive *Arabidopsis thaliana* and salt-tolerant *Thellungiella salsuginea*. Plant Sci..

[15] Guan P., Wang J., Li H., Xie C., Zhang S., Wu C., Yang G., Yan K., Huang J., Zheng C. (2018). SENSITIVE TO SALT1, An Endoplasmic Reticulum-Localized Chaperone, Positively Regulates Salt Resistance. Plant Physiol..

[16] Rodriguez M.C., Mehta D., Tan M., Uhrig R.G. (2021). Quantitative Proteome and PTMome Analysis of *Arabidopsis thaliana* Root Responses to Persistent Osmotic and Salinity Stress. Plant Cell Physiol..

[17] Gehring W.J. (1987). Homeo boxes in the study of development. Science.

[18] Kessel M., Gruss P. (1990). Murine developmental control genes. Science.

[19] Vollbrecht E., Veit B., Sinha N., Hake S. (1991). The developmental gene Knotted-1 is a member of a maize homeobox gene family. Nature.

[20] Kerstetter R.A., Laudencia-Chingcuanco D., Smith L.G., Hake S. (1997). Loss-of-function mutations in the maize homeobox gene, knotted1, are defective in shoot meristem maintenance. Development.

[21] Pautot V., Dockx J., Hamant O., Kronenberger J., Grandjean O., Jublot D., Traas J. (2001). KNAT2: Evidence for a link between knotted-like genes and carpel development. Plant Cell.

[22] Lincoln C., Long J., Yamaguchi J., Serikawa K., Hake S. (1994). A knotted1-like homeobox gene in *Arabidopsis* is expressed in the vegetative meristem and dramatically alters leaf morphology when overexpressed in transgenic plants. Plant Cell.

[23] Dean G., Casson S., Lindsey K. (2004). *KNAT6* gene of *Arabidopsis* is expressed in roots and is required for correct lateral root formation. Plant Mol. Biol..

[24] Serikawa K.A., Martinez-Laborda A., Zambryski P. (1996). Three knotted1-like homeobox genes in *Arabidopsis*. Plant Mol. Biol..

[25] Zhao M., Yang S., Chen C.Y., Li C., Shan W., Lu W., Cui Y., Liu X., Wu K. (2015). *Arabidopsis* BREVIPEDICELLUS interacts with the SWI2/SNF2 chromatin remodeling ATPase BRAHMA to regulate *KNAT2* and *KNAT6* expression in control of inflorescence architecture. PLoS Genet..

[26] Truernit E., Haseloff J. (2007). A Role for KNAT Class II Genes in Root Development. Plant Signal. Behav..

[27] Venglat S.P., Dumonceaux T., Rozwadowski K., Parnell L., Babic V., Keller W., Martienssen R., Selvaraj G., Datla R. (2002). The homeobox gene *BREVIPEDICELLUS* is a key regulator of inflorescence architecture in *Arabidopsis*. Proc. Natl. Acad. Sci. USA.

[28] Koornneef M., van Eden J., Hanhart C.J. (1983). Linkage map of *Arabidopsis thaliana*. J. Hered..

[29] Chuck G., Lincoln C., Hake S. (1996). *KNAT1* induces lobed leaves with ectopic meristems when overexpressed in *Arabidopsis*. Plant Cell.

[30] Felipo-Benavent A., Urbez C., Blanco-Tourinan N., Serrano-Mislata A., Baumberger N., Achard P., Agusti J., Blazquez M.A., Alabadi D. (2018). Regulation of xylem fiber differentiation by gibberellins through DELLA-KNAT1 interaction. Development.

[31] Ben-Targem M., Ripper D., Bayer M., Ragni L. (2021). Auxin and gibberellin signaling cross-talk promotes hypocotyl xylem expansion and cambium homeostasis. J. Exp. Bot..

[32] Xie J., Qi B., Mou C., Wang L., Jiao Y., Dou Y., Zheng H. (2022). *BREVIPEDICELLUS* and *ERECTA* control the expression of *AtPRX17* to prevent *Arabidopsis* callus browning. J. Exp. Bot..

[33] Yokoyama R., Nishitani K. (2001). A comprehensive expression analysis of all members of a gene family encoding cell-wall enzymes allowed us to predict cis-regulatory regions involved in cell-wall construction in specific organs of *Arabidopsis*. Plant Cell Physiol..

[34] Pitaksaringkarn W., Matsuoka K., Asahina M., Miura K., Sage-Ono K., Ono M., Yokoyama R., Nishitani K., Ishii T., Iwai H. (2014). *XTH20* and *XTH19* regulated by ANAC071 under auxin flow are involved in cell proliferation in incised *Arabidopsis* inflorescence stems. Plant J..

[35] Rose J.K., Braam J., Fry S.C., Nishitani K. (2002). The XTH family of enzymes involved in xyloglucan endotransglucosylation and endohydrolysis: Current perspectives and a new unifying nomenclature. Plant Cell Physiol..

[36] Fry S.C., Smith R.C., Renwick K.F., Martin D.J., Hodge S.K., Matthews K.J. (1992). Xyloglucan endotransglycosylase, a new wall-loosening enzyme activity from plants. Biochem. J..

[37] Miedes E., Lorences E.P. (2009). Xyloglucan endotransglucosylase/hydrolases (XTHs) during tomato fruit growth and ripening. J. Plant Physiol..

[38] Nishitani K., Tominaga R. (1992). Endo-xyloglucan transferase, a novel class of glycosyltransferase that catalyzes transfer of a segment of xyloglucan molecule to another xyloglucan molecule. J. Biol. Chem..

[39] Jiang Y., Li Y., Lu C., Tang Y., Jiang X., Gai Y. (2020). Isolation and characterization of *Populus* xyloglucan endotransglycosylase/hydrolase (XTH) involved in osmotic stress responses. Int. J. Biol. Macromol..

[40] Cheng Z., Zhang X., Yao W., Gao Y., Zhao K., Guo Q., Zhou B., Jiang T. (2021). Genome-wide identification and expression analysis of the xyloglucan endotransglucosylase/hydrolase gene family in poplar. BMC Genom..

[41] Zhu X.F., Lei G.J., Wang Z.W., Shi Y.Z., Braam J., Li G.X., Zheng S.J. (2013). Coordination between apoplastic and symplastic detoxification confers plant aluminum resistance. Plant Physiol..

[42] Zhu X.F., Wan J.X., Sun Y., Shi Y.Z., Braam J., Li G.X., Zheng S.J. (2014). Xyloglucan Endotransglucosylase-Hydrolase17 Interacts with Xyloglucan Endotransglucosylase-Hydrolase31 to Confer Xyloglucan Endotransglucosylase Action and Affect Aluminum Sensitivity in *Arabidopsis*. Plant Physiol..

[43] Cho S.K., Kim J.E., Park J.A., Eom T.J., Kim W.T. (2006). Constitutive expression of abiotic stress-inducible hot pepper *CaXTH3*, which encodes a xyloglucan endotransglucosylase/hydrolase homolog, improves drought and salt tolerance in transgenic *Arabidopsis* plants. FEBS Lett..

[44] Choi J.Y., Seo Y.S., Kim S.J., Kim W.T., Shin J.S. (2011). Constitutive expression of *CaXTH3*, a hot pepper xyloglucan endotransglucosylase/hydrolase, enhanced tolerance to salt and drought stresses without phenotypic defects in tomato plants (*Solanum lycopersicum cv. Dotaerang*). Plant Cell Rep..

[45] Han Y., Wang W., Sun J., Ding M., Zhao R., Deng S., Wang F., Hu Y., Wang Y., Lu Y. (2013). *Populus euphratica* XTH overexpression enhances salinity tolerance by the development of leaf succulence in transgenic tobacco plants. J. Exp. Bot..

[46] Han Y., Han S., Ban Q., He Y., Jin M., Rao J. (2017). Overexpression of persimmon *DkXTH1* enhanced tolerance to abiotic stress and delayed fruit softening in transgenic plants. Plant Cell Rep..

[47] Hou X., Li L., Peng Z., Wei B., Tang S., Ding M., Liu J., Zhang F., Zhao Y., Gu H. (2010). A platform of high-density INDEL/CAPS markers for map-based cloning in *Arabidopsis*. Plant J..

[48] Chang C.P., Jacobs Y., Nakamura T., Jenkins N.A., Copeland N.G., Cleary M.L. (1997). Meis proteins are major in vivo DNA binding partners for wild-type but not chimeric Pbx proteins. Mol. Cell Biol..

[49] Xu P., Fang S., Chen H., Cai W. (2020). The brassinosteroid-responsive xyloglucan endotransglucosylase/hydrolase 19 (*XTH19*) and *XTH23* genes are involved in lateral root development under salt stress in *Arabidopsis*. Plant J..

[50] Stratilova B., Kozmon S., Stratilova E., Hrmova M. (2020). Plant Xyloglucan Xyloglucosyl Transferases and the Cell Wall Structure: Subtle but Significant. Molecules.

[51] Frangedakis E., Saint-Marcoux D., Moody L.A., Rabbinowitsch E., Langdale J.A. (2017). Nonreciprocal complementation of KNOX gene function in land plants. New Phytol..

[52] Sakakibara K., Ando S., Yip H.K., Tamada Y., Hiwatashi Y., Murata T., Deguchi H., Hasebe M., Bowman J.L. (2013). *KNOX2* genes regulate the haploid-to-diploid morphological transition in land plants. Science.

[53] Woerlen N., Allam G., Popescu A., Corrigan L., Pautot V., Hepworth S.R. (2017). Repression of *BLADE-ON-PETIOLE* genes by KNOX homeodomain protein BREVIPEDICELLUS is essential for differentiation of secondary xylem in *Arabidopsis* root. Planta.

[54] Mele G., Ori N., Sato Y., Hake S. (2003). The knotted1-like homeobox gene *BREVIPEDICELLUS* regulates cell differentiation by modulating metabolic pathways. Genes Dev..

[55] Hay A., Barkoulas M., Tsiantis M. (2006). ASYMMETRIC LEAVES1 and auxin activities converge to repress BREVIPEDICELLUS expression and promote leaf development in *Arabidopsis*. Development.

[56] Hrmova M., Farkas V., Lahnstein J., Fincher G.B. (2007). A Barley xyloglucan xyloglucosyl transferase covalently links xyloglucan, cellulosic substrates, and (1,3;1,4)-β-D-glucans. J. Biol. Chem..

[57] Jaspers P., Kangasjarvi J. (2010). Reactive oxygen species in abiotic stress signaling. Physiol. Plant..

[58] Clough S.J., Bent A.F. (1998). Floral dip: A simplified method for Agrobacterium-mediated transformation of *Arabidopsis thaliana*. Plant J..

[59] Langmead B., Trapnell C., Pop M., Salzberg S.L. (2009). Ultrafast and memory-efficient alignment of short DNA sequences to the human genome. Genome Biol..

[60] Li G., Siddiqui H., Teng Y., Lin R., Wan X.Y., Li J., Lau O.S., Ouyang X., Dai M., Wan J. (2011). Coordinated transcriptional regulation underlying the circadian clock in *Arabidopsis*. Nat. Cell Biol..

[61] Cheng Z.J., Zhao X.Y., Shao X.X., Wang F., Zhou C., Liu Y.G., Zhang Y., Zhang X.S. (2014). Abscisic acid regulates early seed development in *Arabidopsis* by ABI5-mediated transcription of *SHORT HYPOCOTYL UNDER BLUE1*. Plant Cell.

[62] Yan J., Huang Y., He H., Han T., Di P., Sechet J., Fang L., Liang Y., Scheller H.V., Mortimer J.C. (2019). Xyloglucan endotransglucosylase-hydrolase30 negatively affects salt tolerance in *Arabidopsis*. J. Exp. Bot..

[63] Nan N., Wang J., Shi Y., Qian Y., Jiang L., Huang S., Liu Y., Wu Y., Liu B., Xu Z.Y. (2020). Rice plastidial NAD-dependent malate dehydrogenase 1 negatively regulates salt stress response by reducing the vitamin B6 content. Plant Biotechnol. J..

