# Supplementary material for: BREVIPEDICELLUS Positively Regulates Salt-Stress Tolerance in Arabidopsis thaliana
Source: Int J Mol Sci. 2023 Jan 5;24(2):1054. doi: 10.3390/ijms24021054 (PMC9866879; doi:10.3390/ijms24021054)
Supplement: Supplementary file 1 [file ijms-24-01054-s001.zip › Supplementary Figures.pdf]

Figure S1

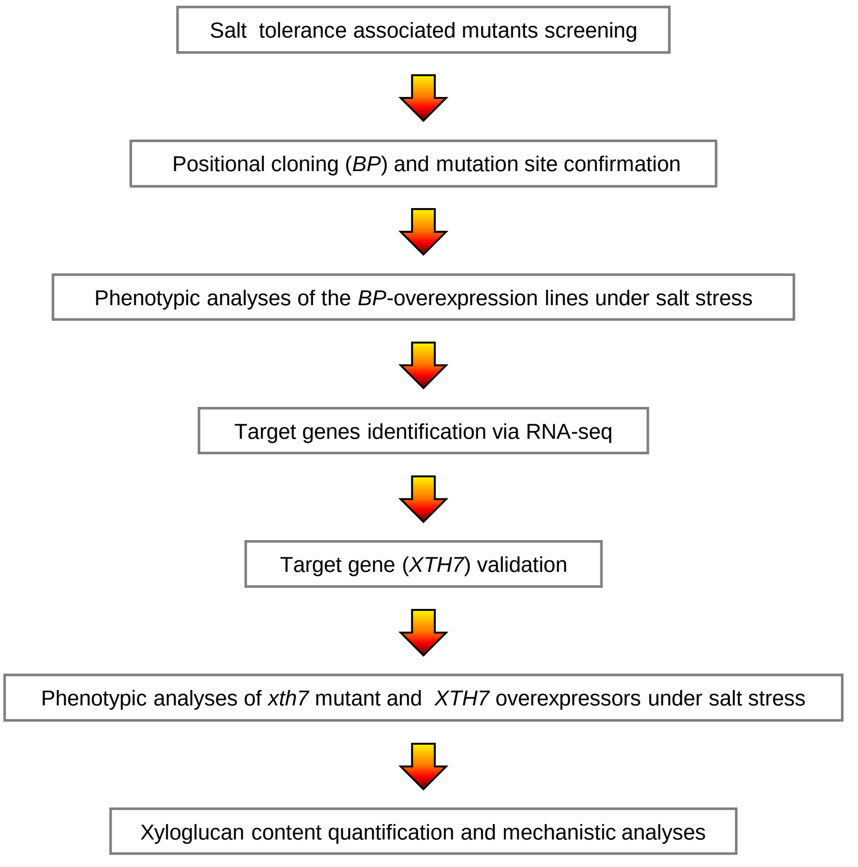

Figure S1. A framework of this study.

Figure S2

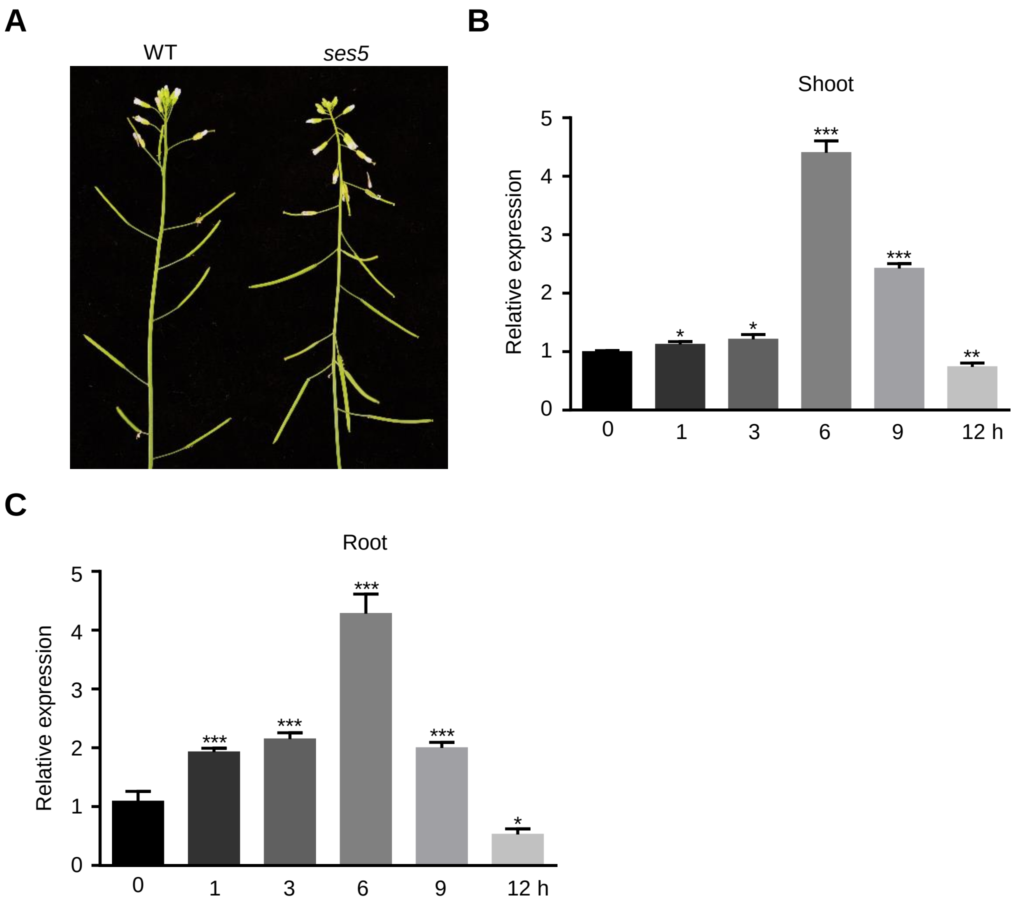

Figure S2. Characterization of *ses5* and expression pattern of *BP.* (**A**) Close-ups of floral nodes with siliques from WT and *ses5*. (**B**,**C**) Responsiveness of *BP* to salt stress. RNA was extracted from 7-day-old WT seedlings treated with 200 mM NaCl for 0, 1, 3, 6, 9 and 12 h. The data were normalized against the expression of *GAPDH* and *UBQ10*. The means were calculated from three independent replicates and compared with the no-treatment condition (0 h). The bars indicate means ± sd of three independent replicates. *, $P < 0.05$, **, $P < 0.01$, ***, $P < 0.001$ (Student's *t* test).

Figure S3

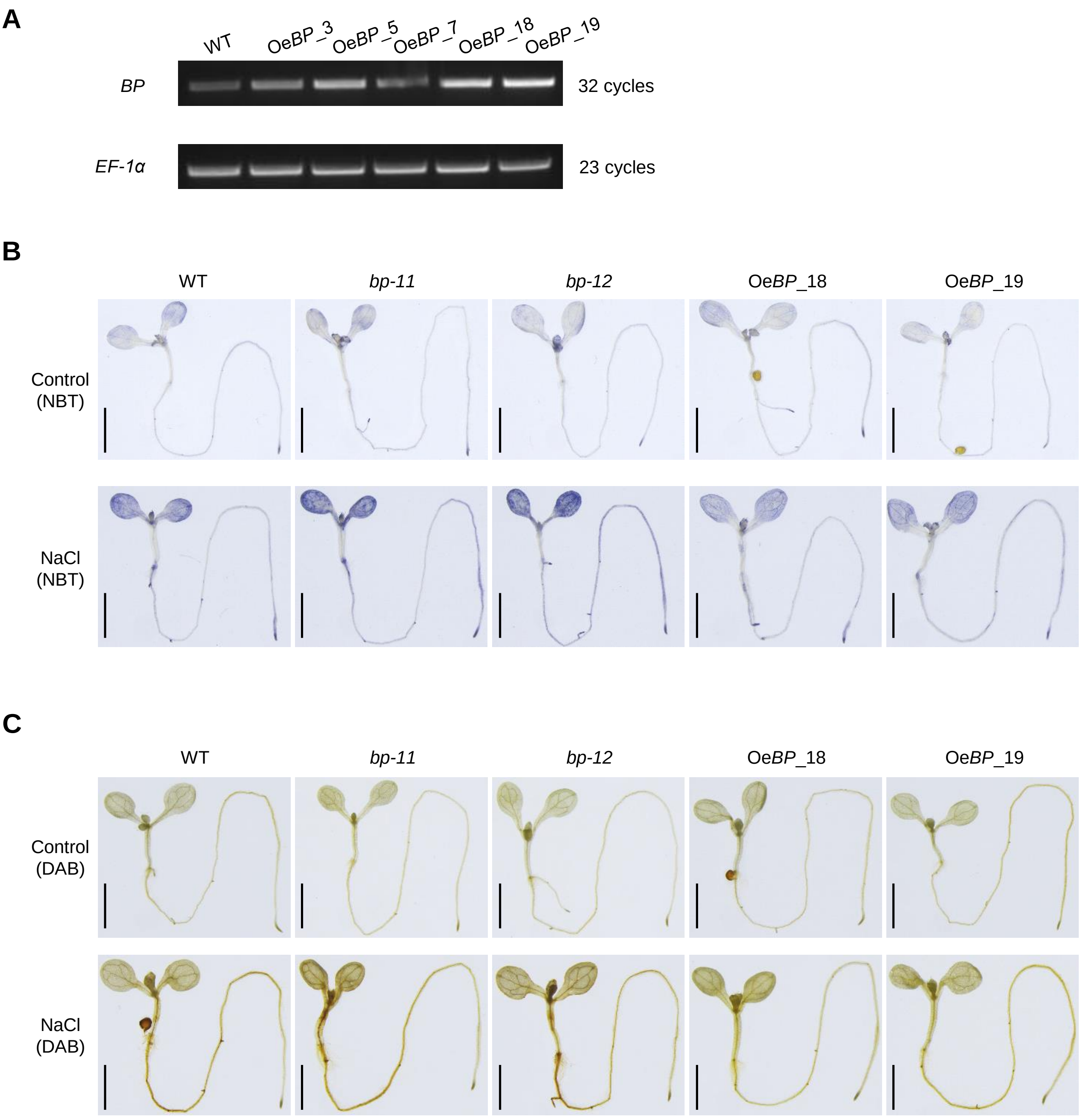

Figure S3. RT-PCR analysis of the *BP* transcripts and ROS levels in the WT, mutants and overexpression lines plants. (**A**) RT-PCR analysis of the *BP* transcripts in the WT and *BP*-overexpression lines. *EF-1α* was used as control. (**B**) NBT staining of O_2^- in 7-day-old WT, mutants and overexpression lines plants exposed to either 0 mM (Control) (top panel) or 150 mM NaCl for 24 h (bottom panel). (**C**) DAB staining of H_2O_2 in 7-day-old WT, mutants and overexpression lines plants exposed to either 0 mM (Control) (top panel) or 150 mM NaCl for 24 h (bottom panel). Scale bar = 5 mm.

Figure S4

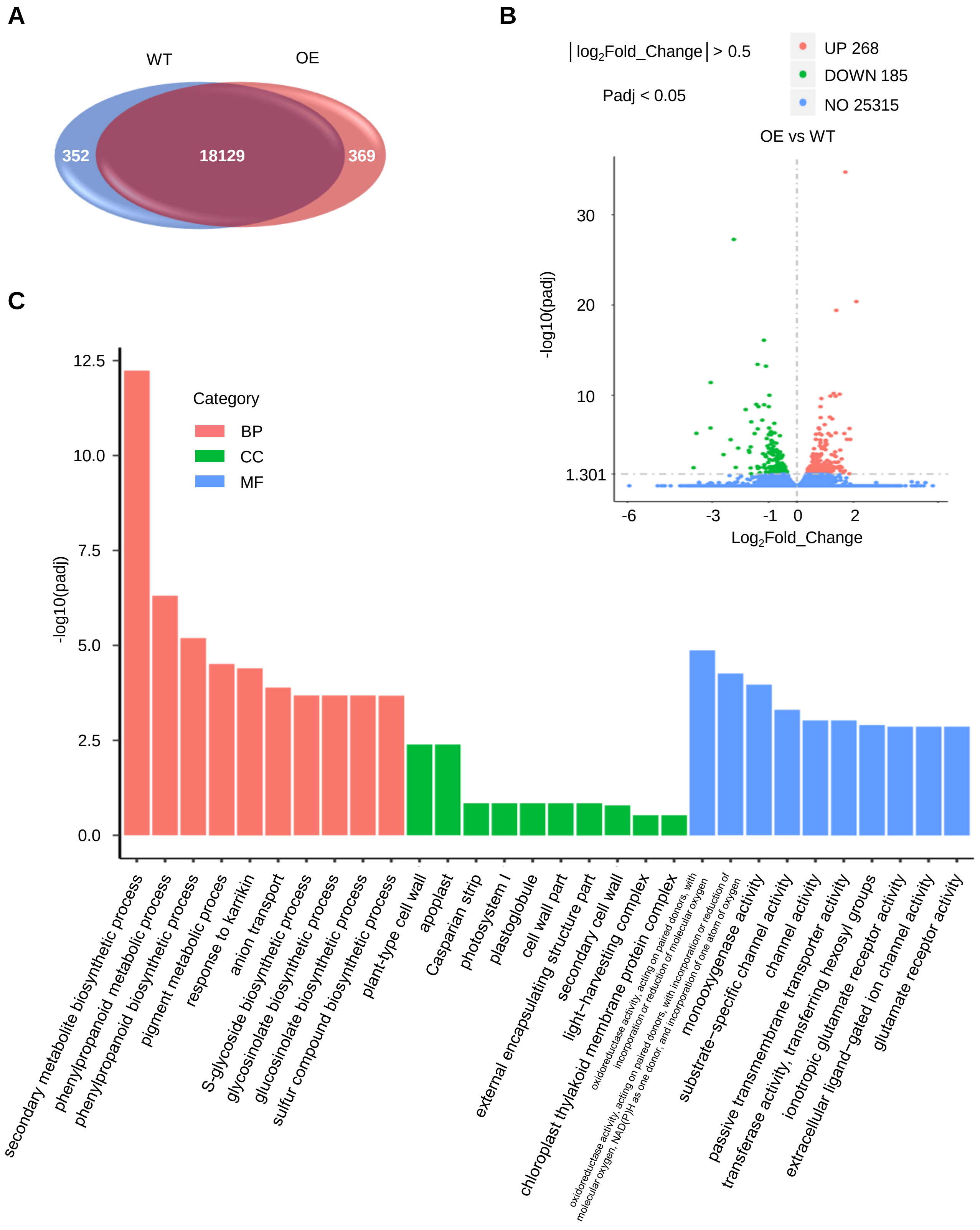

Figure S4. RNA-seq analyses. (**A**) Venn diagram of the differentially expressed genes (DEGs) in WT and *BP*-overexpression line (Oe*BP*_18). (**B**) Significance analysis of the DEGs in WT and *BP*-overexpression line. (**C**) GO enrichment analysis of DEGs. BP, biological process; CC, cellular component; MF, molecular function.

Figure S5

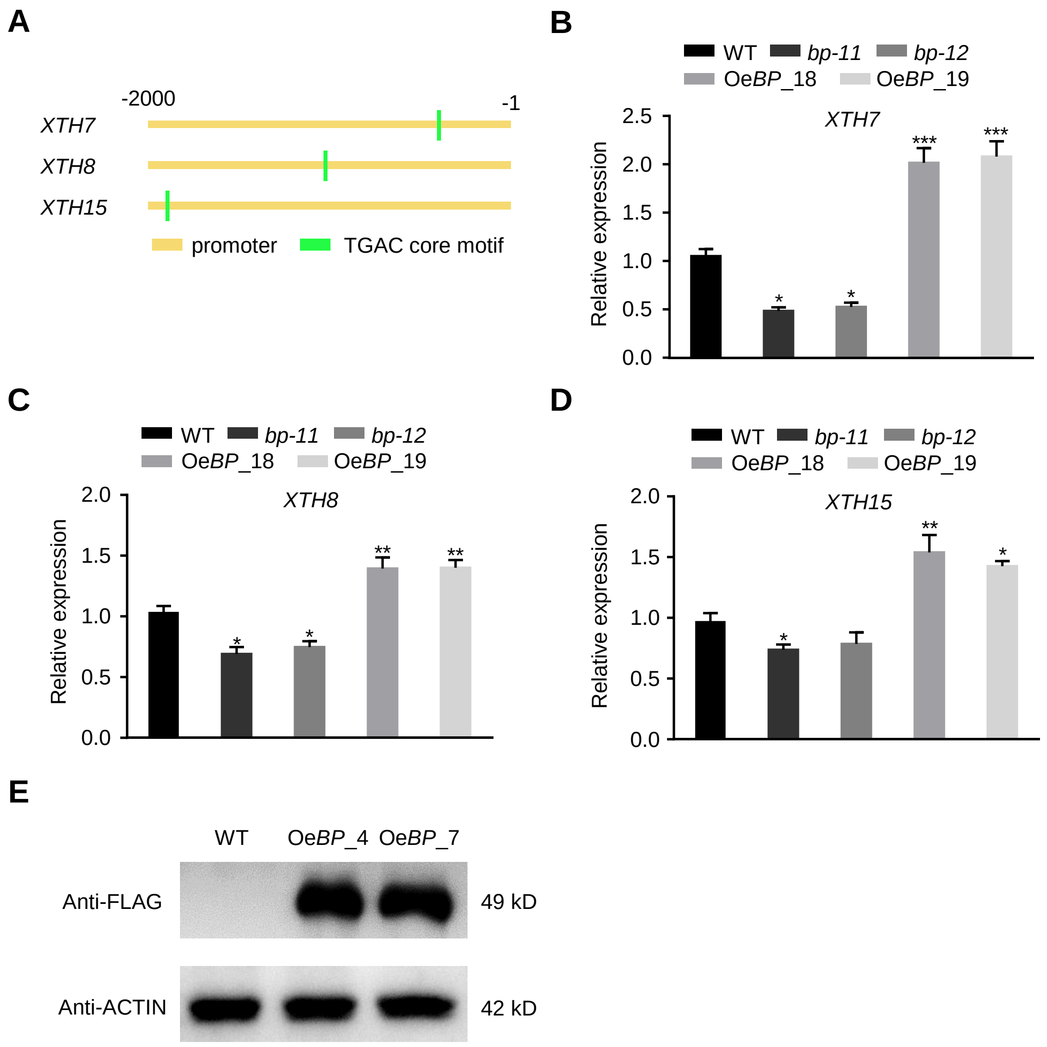

Figure S5. Expression analyses of *XTHs*. (**A**) Diagrams of the promoters. (**B**-**D**) The expression level of *XTH7*, *XTH8* and *XTH15* in the WT, *bp-11*, *bp-12*, Oe*BP*_18 and Oe*BP*_19 plants. The data were normalized against the expression of *GAPDH* and *UBQ10*. The bars indicate means ± sd of three independent replicates. *, $P < 0.05$, **, $P < 0.01$, ***, $P < 0.001$ (Student's *t* test). (**E**) The protein level of BP in transgenic lines (Oe*BP*_4 and Oe*BP*_7). Seven-day-old seedlings were used for protein extraction. BP was detected by anti-FLAG antibody. Anti-ACTIN antibody was used to quantify actin (loading control).

Figure S6

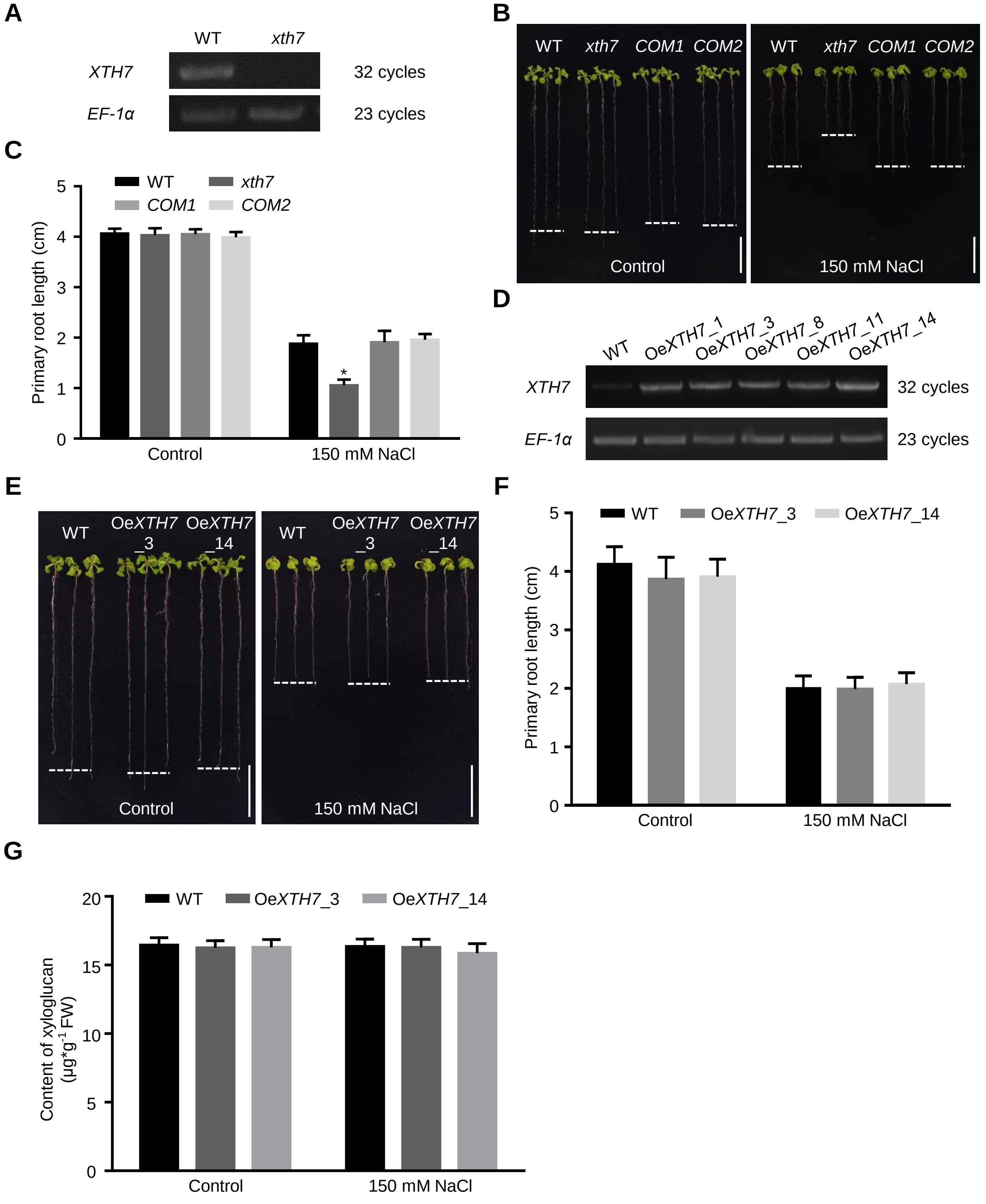

Figure S6. Phenotype analysis of *xth7* and *XTH7*-overexpression lines. (**A**) RT-PCR analysis of the *XTH7* transcripts in the WT and *xth7* plants. *EF-1α* was used as control. (**B**) Photographs of WT, *xth7* and complementary lines (*COM1* and *COM2*) seedlings grown vertically on control or salt-containing medium. Three-day-old seedlings grown on 1/2 MS medium were transferred to 1/2 MS medium with or without 150 mM NaCl for another 10 d. Scale bar = 1 cm. (**C**) Primary root length of the seedlings in B. The bars indicate means ± sd of three independent replicates. (**D**) RT-PCR analysis of the *XTH7* transcripts in the WT and *XTH7*-overexpression lines. *EF-1α* was used as control. (**E**) Photographs of WT and *XTH7*-overexpression seedlings grown vertically on control or salt-containing medium. Three-day-old seedlings grown on 1/2 MS medium were transferred to 1/2 MS medium with or without 150 mM NaCl for another 10 d. Scale bar = 1 cm. (**F**) Primary root length of the seedlings in E. The bars indicate means ± sd of three independent replicates. (**G**) Extractable xyloglucan content of WT and *XTH7*-overexpression lines by iodine staining with or without 150 mM NaCl treatment. The bars indicate means ± sd of three independent replicates. *, $P < 0.05$, (Student's *t* test).

Figure S7

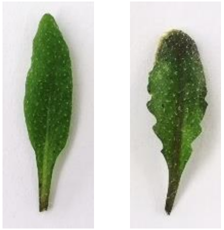

Figure S7. Representative rosette leaves of WT and *BP*-overexpression line (Oe*BP*_18).

Figure S8

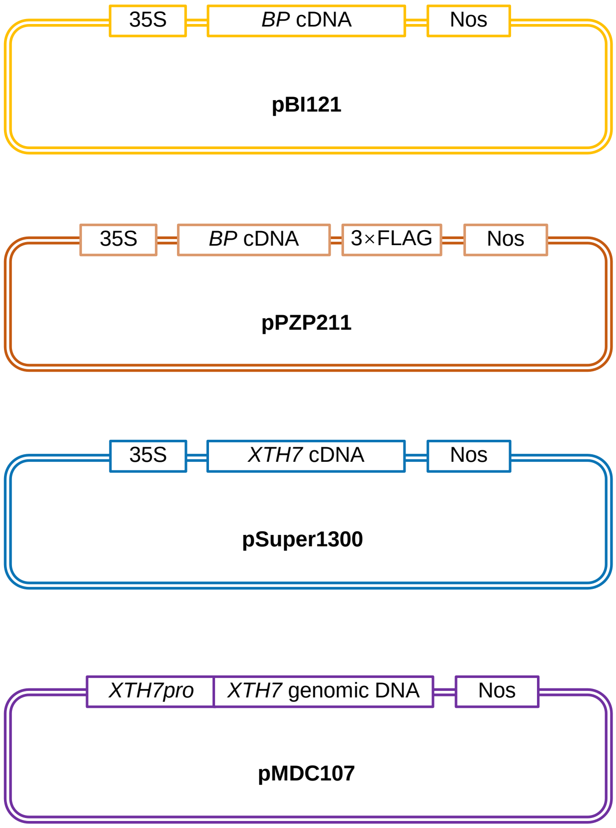

Figure S8. Vector constructs used in this study.
