# Supplementary material for: BREVIPEDICELLUS Positively Regulates Salt-Stress Tolerance in Arabidopsis thaliana
Source: Int J Mol Sci. 2023 Jan 5;24(2):1054. doi: 10.3390/ijms24021054 (PMC9866879; doi:10.3390/ijms24021054)
Supplement: Supplementary file 1 [file ijms-24-01054-s001.zip › Supplementary Table S2.pdf]

Table S2 List of primers used in this study

Prime Name	Primer sequence (from 5′ to 3′)
BP-pBI121-F	GAATTCATGGAAGAATACCAGCATGACAACAGC
BP-pBI121-R	GTCGACGACCGAGACGATAAGGTCCATCA
BP-pPZP211-F	GGTACCATGGAAGAATACCAGCATGACAACAGC
BP-pPZP211-R	GTCGACGACCGAGACGATAAGGTCCATCA
BP-RT-F	ATCCTGATGGGAAGAGTGACA
BP-RT-R	TGCAGACCATCCATCACCATG
BP-pGBKT7-F	GAATTCATGGAAGAATACCAGCATGACAACAGC
BP-pGBKT7-R	GGATCCGACCGAGACGATAAGGTCCATCA
BP-pJG4-5-F	TTATGCCTCTCCCGAATTCATGGAAGAATACCAGCATGACAACAGC
BP-pJG4-5-R	AGAAGTCCAAAGCTTCTCGAGTTATGGACCGAGACGATAAGGTC
BP-pGEX4T-3-F	GGATCCATGGAAGAATACCAGCATGACAACAGC
BP-pGEX4T-3-R	GTCGACTGGACCGAGACGATAAGGTC
XTH7-pSuper1300-F	GTCGACATGGTGGTGTCACTCTTCTCTTCC
XTH7-pSuper1300-R	GGTACCGATTCCAGCCGAGCATTCTGG
XTH7-pMDC107-F	GCAGGTCGACTCTAGAATAGTTCTTACTTCCATGTGGATTTGT
XTH7-pMDC107-R	CCCCCTCGAGGCGCGCCTCAGATTCCAGCCGAGCATTC
XTH7-pLacZi2μ-F	CAATGTAATGCTGACACGTGTCCCAG
XTH7-pLacZi2μ-R	TCGACTGGGACACGTGTCAGCATTACATTGGTAC
XTH7-pGreenII0800-F	TCGACATGTAATGCTGACACGTGTCCCAGTAG
XTH7-pGreenII0800-R	GATCCTACTGGGACACGTGTCAGCATTACATG
XTH7-Probe-F	TGTAATGCTGACACGTGTCCCAGT
XTH7-Probe-R	ACTGGGACACGTGTCAGCATTACA
XTH7-mProbe-F	TGTAATGCAAAAACGTGTCCCAGT
XTH7-mProbe-R	ACTGGGACTTTTACGTGCATTACA
XTH7-qRT-F	TAGACAATGTGCCCATCAGGGT
XTH7-qRT-R	GGTGGAACAGGAAAACGAGATTT
XTH8-qRT-F	ATGGAGACGGAAAGGAGGATCA
XTH8-qRT-R	AGCCCAAGGGCTAATGGAACAC
XTH15-qRT-F	ATGGGTCCAAGTTCGAGCCTC
XTH15-qRT-R	GACTCTGGACTTCTTGCATTCTGGAG
SALK_201184-F	CTCTTATTACATGGCCCATGG
SALK_201184-R	TACATACATTTGCCTGCATGC
EF-1α-F	GTATGGTTGTTACCTTTCCTCCCACAG
EF-1α-R	CATCATTTGGCACCCTTCTTCACTGC
UBQ10-F	GCCAAGATCCAGGACAAGG
UBQ10-R	CGCAGGACCAAGTGAAGAG
GAPDH-F	TTGGTGACAACAGGTCAAGCA
GAPDH-R	AAACTTGTCGCTCAATGCAATC
LBb1.3	ATTTTGCCGATTTCGGAAC
T23J18-F	GATATTTGTTTTGCTAACAC
T23J18-R	TAATAAAGTTCCAGCTTTGA

T22J18-F	CACTGCAACAAAGTGGAAAT
T22J18-R	ATCCGTTTCAATATCCACAA
F28H19-F	TGCGGGAGTGTGATAGAATA
F28H19-R	TCCTCGAAAGATTCATTGA
F19K23-F	GAATTCTGTAACATCCCATTTCC
F19K23-R	GGTCTAATTGCCGTTGTTGC
T17F3-F	GGACCGACGGTTACGAGAGT
T17F3-R	TAACGGGCCGTTGCAAGA
T23K3-F	CGTGTTTACCGGGTCGGA
T23K3-R	AAAACCCTTGAAGAATACG
T12H3-F	TAGTCTGAGCTTACCAATA
T12H3-R	TTACCCTCGACTCGTAAC
F3P11-F	ATGTATTTGTTGCAAAATAA
F3P11-R	TGCACAGAAGAAAAAACTA
T20P8-F	TCCGATTCGATTAAACTC
T20P8-R	TTATTTCCTATTTCAAGACT
T16B24-F	ATGAACGGAGTAGCTATC
T16B24-R	CGCGTAGAACATAATCTGTA
F20H23-F	CAATGGGAAGAAGGTGTGAG
F20H23-R	CGCATTTCCATAAGTTTGTT
MGL6-F	ACCTGTTCAGTCTATGTTAC
MGL6-R	GGGAATTATTAACATTATCA
K1G2-F	ATGAGCTTTAGGAGTGTGTA
K1G2-R	AATTTTGTCCCAAAAGAATA
T32N15-F	CAAAAGAAATGCAACGAGAC
T32N15-R	TTTGATCATGAATGGTAGTG
T26I12-F	GAGCAACATTAAGGATAGAA
T26I12-R	ATCTCATACTCATAATATGTAG
T4I9-F	TTATAGCAAACGTACAAGTC
T4I9-R	CTGCATACACGTCGTCTC
F17A8-F	CTGGACCCTAGTGGATGT
F17A8-R	GACGGTTCTCCATTAATTAT
FCA8-F	TTCGGAGAAAGAAACGACAT
FCA8-R	ATGGAACTATTCAGGCATTA
T15N24-F	GCAACCGCTGCTGCTTTA
T15N24-R	AATATTTGGCTTTGCGTAGA
T4L20-F	ACCCTAAAACAATGTCTCTT
T4L20-R	TGCTAACATGGAAATTTGTC
MBK20-F	CTCTGTTGGGGCAAAACC
MBK20-R	CTCTGTTGGGGCAAAACC
MYJ24-F	TTCATGAGAGCGGCATTC
MYJ24-R	TTCATGAGAGCGGCATTC
T26D22-F	CACAGGCCATTGGATGTA
T26D22-R	TGTTAGAACCCACCATTTG

K6M13-F	CCTGTTCCAATGAATATG
K6M13-R	TGTAGCTGCTGAGTTGTC
MQB2-F	AAAAGGCGACTACTAGCA
MQB2-R	GCCATTTATTTGGTCAAC
F14G16-F	CTCCGATGAATCACCATTAG
F14G16-R	CCGGTTTGGCTTGTCTGTTC
T3E15-F	GCTTAATACCAACGTTTTCC
T3E15-R	GCTGAAGAGAGAGTTAGCTC
F1K3-F	CCGATCGCCAAGAAGTAA
F1K3-R	GTGCAAACACTATCCAAAAT
F9M13-F	TTGGAGGAGTCATGAAGTTG
F9M13-R	TTGTAAGTAAAGTAACCTCTCT
T15G18-F	GAAGCCAAATCGTCTGTT
T15G18-R	CGAGGATTAGTAGGCTATA
T3F12-F	ATCCTTCTCGCTCTCCTGTG
T3F12-R	TGTTGACGCCTTGCCATA
T15F16-F	TCATTGATGTGTGTGTGGGTG
T15F16-R	TGGGATTGTTGATTTCACCACA
